# Supplementary material for: Tead1a Initiates Transcriptional Priming Through the TEAD1a/YAP‐Notch1‐Spi1/Cebpα Axis to Promote Neutrophil Fate
Source: Adv Sci (Weinh). 2025 Aug 28;12(41):e05441. doi: 10.1002/advs.202505441 (PMC12591127; doi:10.1002/advs.202505441)
Supplement: Supplementary file 4 — Supplementary Dataset [file ADVS-12-e05441-s004.zip › 3-4.zip!/3-4/02-4.cluster/cluster11/Enrich/ego_CC.pdf]

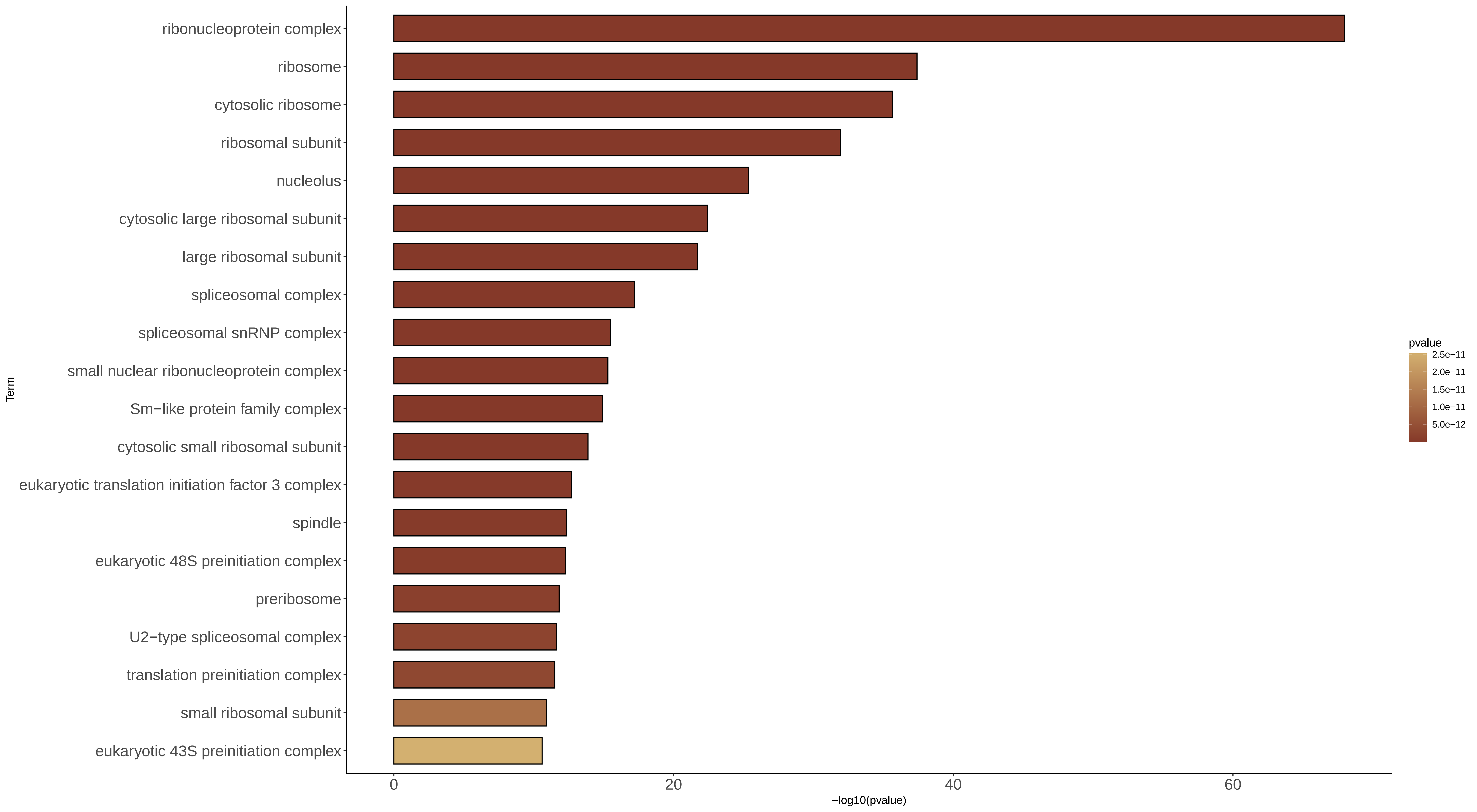
ribonucleoprotein complex
ribosome
cytosolic ribosome
ribosomal subunit
nucleolus
cytosolic large ribosomal subunit
large ribosomal subunit
spliceosomal complex
spliceosomal snRNP complex
small nuclear ribonucleoprotein complex
Sm−like protein family complex
cytosolic small ribosomal subunit
eukaryotic translation initiation factor 3 complex
spindle
eukaryotic 48S preinitiation complex
preribosome
U2−type spliceosomal complex
translation preinitiation complex
small ribosomal subunit
eukaryotic 43S preinitiation complex
Term
0
20
40
60
−log10(pvalue)
pvalue
2.5e−11
2.0e−11
1.5e−11
1.0e−11
5.0e−12
